# Genomics in Diabetic Kidney Disease: A 2024 Update

**DOI:** 10.2174/0113892029300247240325080421

**Published:** 2024-04-02

**Authors:** Stefanos Roumeliotis, Maria Divani, Eleni Stamellou, Vassilios Liakopoulos

**Affiliations:** 1 Second Department of Nephrology, School of Medicine, AHEPA Hospital, Aristotle University of Thessaloniki, Thessaloniki, Greece;; 2 Department of Nephrology, Faculty of Medicine, School of Health Sciences, University of Thessaly, Larissa, Greece;; 3 Department of Nephrology, Medical School, University Hospital of Ioannina, Ioannina, Greece

**Keywords:** Chronic kidney disease, diabetic kidney disease, end-stage kidney disease, genomics, GWAS, single nucleotide polymorphisms

## Abstract

Diabetic Kidney Disease (DKD) remains the leading cause of Chronic and End Stage Kidney Disease (ESKD) worldwide, with an increasing epidemiological burden. However, still, the disease awareness remains low, early diagnosis is difficult, and therapeutic management is ineffective. These might be attributed to the fact that DKD is a highly heterogeneous disease, with disparities and variability in clinical presentation and progression patterns. Besides environmental risk factors, genetic studies have emerged as a novel and promising tool in the field of DKD. Three decades ago, family studies first reported that inherited genetic factors might confer significant risk to DKD development and progression. During the past decade, genome-wide association studies (GWASs) screening the whole genome in large and multi-ethnic population-based cohorts identified genetic risk variants associated with traits defining DKD in both type 1 and 2 diabetes. Herein, we aim to summarize the existing data regarding the progress in the field of genomics in DKD, present how the revolution of GWAS expanded our understanding of pathophysiologic disease mechanisms and finally, suggest potential future directions.

   Diabetic Kidney Disease (DKD) remains the leading cause of Chronic (CKD) and End Stage Kidney Disease (ESKD) worldwide, with increasing epidemiological burden. DKD is a clinical diagnosis based upon the presence of persistent albuminuria and/or persistent reduced estimated glomerular filtration rate (eGFR), or both, in patients with diabetes. This definition is based on clinical features and does not actually indicate the exact pathological phenotype of kidney damage due to diabetes, but highlights that DKD is a highly heterogeneous disease that can be presented in both the traditional albuminuric or non-albuminuric pattern, with disparities and variability in clinical presentation and progression involving complex, overlapping pathophysiologic pathways. This might be only partially explained by environmental risk factors. Family studies from three decades ago first showed clustering and aggregation of DKD among type 1 diabetes (T1D) siblings [1]. The heritability estimates ranged from 30% to 75%, depending on the trait and reached higher values for the more severe clinical DKD definitions [2].

   Following the analysis of the whole human Genome in 2004, early genetic studies were performed to investigate candidate genes for DKD, but their results were inconclusive and contradictory due to several limitations: small sample, which led to underpowered studies, lenient statistical thresholds, no external replication and homogenous populations of only one race and ethnicity [3]. Due to these limitations, these studies were prone to both type I (potential false-positive) and type II errors (false-negative). This is why previously reported genetic associations for DKD from early genetic studies failed to be replicated in larger, homogenous populations [3]. This failure highlighted the need for rigorous methodologies in genetic studies applying stringent statistical thresholds, enrolling diverse populations and different ethnic groups and large sample sizes. Moreover, meta-analyses of all available data might also maximize the power, improve the generalizability of findings and contribute to a comprehensive understanding of DKD.

   To overcome these limitations, genome-wide association studies (GWASs) emerged as novel useful tools to identify -in an unbiased way- genetic risk factors for DKD owing to T1D and type 2 diabetes mellitus (T2DM). These studies were actually mapping methods screening the whole genome and were commonly performed on large scales, thus resulting in adequate sample size and statistical power. The large transethnic GWAS and meta-analysis by the Family Investigation of Nephropathy and Diabetes (FIND) was the first to show a uniform risk of new genes for DKD with consistent results across all races and ethnicities [4]. By combining data from 11,847 T1D subjects of European cohort studies, the Genetics of Nephropathy consortium was the first to perform GWAS meta-analysis and show that risk variants in the *AFF3, RGMA* and *MCTP2* genes increased the risk for ESKD [5]. Until today, the largest GWAS in T1D patients was conducted by the Diabetic Nephropathy Collaborative Research Initiative (DNCRI) consortium, in approximately 20,000 T1D subjects from 17 European descent cohorts and showed that a common missense genetic variant of the collagen type 4 alpha 3 chain gene (*COL4AE*) r55703767/Asp326Tyr played a protective role (21% decreased risk for DKD) and was associated with thinner glomerular basement membrane (GBM) [6]. Commonly, the impact of risk variants is exerted through over- or underexpression of the function of causal genes. However, this was the first study to show that this genetic variant affected the product (*i.e*., the thickness of GBM) and not the expression levels of the gene. To overcome selection bias, the researchers used 10 different case-control phenotype definitions of DKD based on different traits (kidney function or albuminuria, or both or ESKD) and found that the effect of *COL4AE* on DKD was significantly stronger in patients with poor glycemic control (HbA1c>7.5%). Since hyperglycemia amplified the effect of *COL4AE* on DKD risk, the authors hypothesized that this gene effect was highly specific to diabetes.

   Although numerous loci have been identified to be associated with DKD, their functional roles on molecular pathways and disease progression still remain unknown. To address this issue, several researchers performed functional studies. Xue *et al.* reported that genes that conferred risk T2DM, were associated with insulin production and β cell sensitivity to lipotoxicity [7], whereas the polymorphisms of ADIPOQ and ACACB were associated with lipid metabolism, TGF-β1 and ELMO1 with inflammation and OS, ACE1/D and AGTR1 with the RAAS system, VEGFA and EPO with angiogenesis, GCKR and TCF7L2 with glucose metabolism and FRMD3 and SHROOM3 with renal structure and function [8] and TRIM27 and HLA-A with immune response and autophagy [9].

   Tziastoudi *et al.* [10] investigated a large number of genes and SNPs (606 variants located in 228 genes, derived from 360 GASs) in a comprehensive and concise systematic review and meta-analysis which made comparisons between healthy controls and DN, diabetic controls and DN and healthy *versus* diabetics *versus* DN cases, in order to distinguish risk variants that might drive DKD progression independently of diabetic milieu. This analysis showed that gene polymorphisms were associated with overexpression of various molecular pathways underlying DKD, including pyruvate metabolism, renin-angiotensin-aldosterone (RAAS) system, inflammation, oxidative stress (OS), lipid metabolism, endothelium function and glucose transport. These findings not only confirmed the clinical utility of drug agents traditionally used for the management of DN (RAAS inhibitors, sodium glucose transport protein 2, statins) but also suggested novel potential therapeutic targets, including OS and inflammation.

   In 860 T2DM patients with diabetic nephropathy (DN) and 650 T2DM patients with normal kidney function and no albuminuria, the GR-DIAGENES consortium found several novel risk variants for DN implicated in the molecular pathway of OS that had never been studied before [11]. Most importantly, after a long follow-up of 7 years, a novel locus of the human soluble epoxide hydrolase (rs11780592) was found to be associated with oxidized LDL and clinical hard endpoints including mortality and CV events, irrespectively of various traditional risk factors [12], thus highlighting the impact of this variant on the molecular pathway of OS and endothelial dysfunction. Moreover, the rs41301097 SNP in the gene encoding cubilin (*CUBN*), an endocytic receptor in the proximal renal tubule, was associated with increased 25(OH)D concentrations [13]. This finding highlighted that another appealing plausibility of genomics in DKD is the identification of responders to a drug therapy, which is important, given the fact that the FDA reported that for every responder to a drug from the top 10 seller list, other 3-24 do not show any clinical benefit. The response to a certain renoprotective therapy for DKD is highly variable according to individuality and practically difficult to predict. Genomics and proteomics might offer an interesting approach to predict treatment responses in DKD. Firstly, individual omics might be used at baseline to classify patients with poor or good responses to a proposed therapy, the “baseline risk prediction” strategy. Another approach is the “dynamic response prediction”, *i.e*., to define as a surrogate endpoint the change in the proteome values after a specific time of proposed therapy and select individuals that are more likely to benefit from subsequent clinical endpoints. These studies are expected to shed light on the pathogenetic mechanisms of DKD and the drug effects. In DKD, omics studies using both baseline and dynamic therapy response prediction are still in their infancy but are very promising. A RCT explored whether the CKD273 proteomic classifier could predict albuminuria response to treatment with spironolactone in a cohort of T2DM patients with resistant hypertension [14], whereas in T2DM patients with micro- or macroalbuminuria, a classifier of 21 serum metabolites was shown to predict albuminuria response to treatment with angiotensin II receptor blockers [15]. Likewise, a nested case-control substudy in the IRMA2 RCT, reported that in patients with DKD (T2DM and microalbuminuria), compared to placebo, treatment with irbesartan over a period of 24 months resulted in significant changes of the urinary peptide classifier CKD273 score. The authors concluded that improvement of fibrosis and collagen turnover might underlie the renoprotective effects of irbesartan [16]. Based on these, a new design of randomized controlled trials (RCTs) was proposed (PRIORITY and SONAR). Instead of blindly enrolling DKD patients in RCTs investigating the effect of a new treatment, we could use genomic and proteomic data to identify those who are more likely to respond and include them as the study population that will be randomized. Finally, the largest GWAS so far included 178,691 diabetics and 1,296,113 non-diabetic controls [17], replicated the results of previous studies for *CUBN* and revealed genes that might inform the development of renoprotective drug agents.

   Apolipoprotein 1 (*APOL1*) SNP has been associated with a 30-fold higher risk for CKD progression and cardiovascular mortality among individuals with African ancestry [18]. However, data from case control and population studies showed that the *APOL1* risk variants might not affect the prevalence but aggravate the progression of DKD [19]. Kidney damage associated with APOL1 risk variants is collectively termed as “APOL1 nephropathies” and includes various forms of focal segmental glomerulosclerosis. The exact mechanism underlying the association between APOL1 SNP and CKD remains unknown. One hypothesis is that APOL1 risk variants might cause mitochondrial injury, while others propose that these variants might have a toxic effect and create holes in the membranes of renal cells, quite similar to how APOL1 opens holes in organelles of the parasite Trypanosoma. However, it has been proposed that APOL1 might influence CKD progression through disturbance in lipid metabolism, progression of atherosclerotic CV disease, induction of cytotoxicity in renal cells and podocytes, change in innate and adaptive immune response and induction of inflammation [20]. The potential clinical utility of genetic analysis for APOL1 lies in the fact that upon diagnosis of CKD, knowing whether a CKD patient has the APOL1 risk variants will lead to early and accurate prediction of CKD progression and help the nephrologist to make therapeutic decisions. This is a quite promising approach and another application of personalized medicine.

   There is also a growing body of evidence suggesting that kidney transplants from *APOL*1 high-risk donors have a higher risk for graft failure compared to non-risk donors, whereas the recipient *APOL1* does not affect the survival outcome of the graft [21]. This natural clinical experiment implies that the topic, kidney-expressed and not circulating *APOL1* is the main culprit in driving *APOL1*-derived CKD. Based on these, the NIH *APOL1* Long-Term Kidney Transplantation Outcomes Network (APOLLO) Consortium is an ongoing study aiming to collect genomic data from over 3,000 kidney donors and 260 transplant programs and investigate the effects of *APOL1* risk variants on kidney transplant outcomes [22]. APOLLO aims to investigate whether the presence of APOL1 high-risk variants in deceased kidney donors is associated with kidney graft survival, decrease in eGFR, and increase in proteinuria after transplantation. The results of this study are expected to provide the rationale for revision of the currently used Kidney Donor Risk Index (KDRI) score and add APOL1 genotype as a part of the formula. Moreover, in everyday clinical practice, the discard of APOL1 low-risk genotype donors might be discouraged, thus leading to increase in transplants and improved outcomes.

   Another interesting genomics initiative is the Kidney Precision Medicine Project, a multicenter prospective study of patients who most commonly will not undergo kidney biopsy for their CKD management (such as Diabetics with CKD). A protocol kidney biopsy will be performed in all patients at enrolment, along with DNA extraction for genetic analysis and analysis of other omics and all patients will be followed for 10 years with clinical hard endpoints, such as CKD progression, ESKD and hospitalizations. Besides building a kidney tissue atlas and a biopsy biobank, this study aims to identify specific groups of patients based on genomics and proteomics (for example, having a certain gene polymorphism) that will present specific clinical outcomes [22]. Most interestingly, besides DKD patients, the researchers will also enroll normoalbuminuric individuals with longstanding type 1 diabetes (for more than 25 years) with preserved normal function. Since the development of DKD needs decades, this group of DKD “resistors” represents the ideal control group for genetic analyses.

   The revolution of GWAS expanded our understanding of genetic susceptibility of DKD but also improved diagnostics, prognosis and therapeutics. Until today, approximately 80 gene loci have been identified as risk factors for DKD (defined as eGFR reduction, albuminuria or both in diabetics) with genome-wide statistical significance. The main challenges of GWASs are follow-up functional studies to establish causality and how to ensure DKD cases and avoid selection bias. Although GWAS studies have revealed numerous SNPs associated with DKD, the translation of these findings into clinical practice remains impractical, due to the fact that individual genetic variants cannot be accounted for the overall risk. To overcome this problem, genetic risk scores (GRSs) have been proposed. A recent systematic review identified 15 studies of GRSs for DKD during the past twenty years and found that the majority of these studies included T2DM patients. The sample size ranged from 687 to 67,403 and the SNPs of interest from 5 to 598 [23]. This review is the first to propose that GRSs might be used in everyday clinical practice, upon diagnosis of diabetes. DNA-analysis and calculation of GRSs might offer tailored, personalized medical surveillance and treatment, in order to prevent DKD. However, DKD is a heterogeneous disease with various pathogenetic mechanisms involved and therefore, a multi-faceted strategy where, besides genetic background, other conditions and co-morbidities are taken into consideration.

   Eight years ago, the former President of the United States, Barack Obama, launched the Precision Medicine Initiative as an emerging approach that would take into account individual variability. Recent advances and novel applications of genomics might offer an unprecedented opportunity for the expansion of Precision Medicine in DKD. The next “big thing” in the prediction of DKD might be the development of new genetic risk scores that will aggregate the effects of several risk variants in one prognostic tool. Moreover, genomic data might also improve the design of RCTs examining intervention strategies and help determine the right dosage of a certain drug for the right patient. To date, genomics has not yet been embedded into everyday clinical practice mainly due to increased cost and limited utility. However, the advances and progress in the genomics field decreased significantly the cost of the whole human genome sequence analysis from 100.000$ in 2000 to 600$ in 2022 (https://www.genome.gov/about-genomics/fact-sheets/Sequencing-Human-Genome-cost).

## CONCLUSION

This rapid and significant reduction of costs will offer clinicians the opportunity to incorporate genetic analysis into routine clinical practice, leading to early identification of DKD, accurate and tailored treatments and paving the way for precision medicine in DKD. Subsequently, this approach will improve the quality of life and health conditions of patients with diabetes. We may not be far from an era of personalized medicine, where genome sequencing might be performed at least once in a diabetic’s lifetime. As we are moving forward to a new era of Precision Medicine in Nephrology, genomics is gaining research interest. Ongoing and future studies are anticipated to shed some light on the field of genomics in DKD.

## LIST OF ABBREVIATIONS

DKD: Diabetic Kidney Disease
eGFR: Estimated Glomerular Filtration Rate
ESKD: End Stage Kidney Disease
GWASs: Genome-wide Association Studies
